# On the Neck as a Medical Region, and on Paroxysmal Paralysis

**Published:** 1849-10

**Authors:** Marshall Hall


*On the Neck as a Medical Region, and on Paroxysmal Paralysis.* By Marshall Hall, M. D., F. R. S., &c.— When I contemplate the nervous, vascular, and muscular structures of the neck, with their relative positions and varied functions, I am astonished at the fact, that in their physiological actions they are never found to interfere materially with each other. I am not the less struck with another fact, that in their pathological conditions these interruptions should so constantly occur, and yet that they have hitherto escaped, in great measure, the observation and attention of physicians.

An athletic person may carry an enormous weight on his head, accurately balanced by the action of the muscles of the neck, without the slightest interference with either the nervous or vascular structures so diffusely and variously spread over this region.

But let *pathological* action, the result of emotion, or excito-motor energy, occur, and then interruption of the course of the blood, and nervous action, the most dire and terrific, ensue.

I have adverted to the nervous, vascular, and muscular structures along the neck; but another element remains to be added; this is the *larynx*, the morbid actions of which, viewed both as effects of preceding causes, or the cause of ulterior effects, are full of the deepest interest.

Nor ought I to omit entirely to notice the pharynx and œsophagus as belonging to this region, and contributing their own influence to the *Class* of morbid phenomena, of which it is the seat.

As remoter organs intimately associated with the pathological actions of the structures in the neck, I must chiefly notice the medulla oblongata and the cerebrum, on the one hand, and the lungs on the other, and especially in their varied relations to comatose, spasmodic, and asphyxial affections.

When the surgeon contemplates the anatomy of the neck, his attention is principally directed to the *arteries*, which may become the subjects of his anxiety and care in practice, whether wounded by accident or diseased by morbid processes, or to be avoided in an operation.

But to the physician the *veins* of the neck are the chief objects of interest. If morbidly compressed, and their blood impeded in its course from the encephalon, many diseases of the utmost moment, are the painful and even terrible result.

The larynx and trachea may be said to present objects of the deepest interest both to the physician and surgeon being equally the seats of disease and of accident, of medical treatment and of surgical operation.

The physician has only to observe the various movements of the eyeball, of the features, of the tongue, of the lower maxilla, of the neck, of the larynx, of the pharynx, &c., to arrive at the conclusion, that in the various spasmodic diseases there is no muscle which may not become, singly or together with others, the subject of spasm.

This principle being admitted, it is only necessary to observe, further, what must be the effect of each of such spasmodic actions, to comprehend the cause and nature of the various symptoms—effects of these—which characterize these diseases.

In estimating the value of this subject, we must bear in mind the principle which has been already enunciated, viz : the difference between the well balanced and harmonious *physiological* actions of the neck, and the abnormal, morbid, and discordant actions of these same muscles in disease, with their *pathological* effects on the subjacent or contiguous structures.

Let us contemplate in this manner the effect of morbid and irregular action of the platysma-myoides, and of the cleido-mastoid and omo-hyoid muscles, on the subjacent external and internal jugular veins respectively.

And shall we leave the arteries and nerves of the neck out of the question, as uninfluenced by these abnormal muscular actions ?

But I can impart no idea of the interest attached to a careful observation of the condition of these *veins*, and thence of the capillary vessels, and of the arteries ; in a word, of the whole *arrière* circulation, in cases of morbid action of the muscles of the neck.

The external jugular and the frontal veins; the color of the cheeks, of the eye and internal eyelid; of the prolabium; the temporal artery; present the phenomena of impeded venous circulation in the most marked degree.

These different points in the cephalic circulation should be examined with the care with which we are wont to examine the conditions of the pulse. The neck should be laid bare, the under eyelid should be everted, the temporal artery should be carefully felt.

One anxious mother could *foretel* the epileptic seizure in her daughter, by observing the fulness of the veins of the neck. One lady observed these veins occasionally to acquire the size of her finger. A medical gentleman drew my attention to the congested condition of the conjunctiva of the under eyelid in his own case. Many patients have presented a cord-like tension of the temporal arteries.

All these phenomena constitute links of the same chain; the first link being compression of the venous trunk by the irregular contraction of the muscle or muscles seated immediately above it; and the last, perhaps a paroxysmal threatening or seizure.

It may be laid down as a principle, that there is no muscle—no set of muscles—in the neck, which may not become spasmodically contracted, and that there is no vein in this region which may not, under the influence of such contraction of muscles, become compressed, and the course of whose blood may not be impeded or arrested. Let us consider the further effect of such compression on the tissues or organs of the head or neck. We shall be led to consider a novel and very interesting question in the pathology of the nervous system. One circumstance must be carefully remembered—the effect of interrupted return of blood along the external jugular is, from its connexions being superficial, far more observable than that of a similar interruption in the internal jugular or vertebral, which can often only be inferred from the symptoms.

I now proceed to consider this subject more especially in regard to certain characteristic affections of the nervous system, which I believe to be at once overlooked, and yet of great importance and prevalence.

We have all heard much of the *tendency* of blood to the head, a condition of the circulation which, in reality, scarcely exists ; and we have scarcely heard of *impeded return* of blood from the head, an event of most frequent, nay, of daily occurrence. In reality, I believe the latter affection has been mistaken for the former.

There is no principle in physiology which could induce or explain tendency of blood to the head. M. Poisseuille has irrefragably shown that the power of the heart is equal in all the bloodvessels of equal size, and of equal distance from the heart. Nothing can influence this force except position, exercise, or muscular effort, or hypertrophy of the heart itself, which may augment the rapidity and force of the circulation ; but then this augmentation of the rapidity of the circulation is general and diffused, like its original force, equally in all the vessels of equal size and form, and at equal distances from the heart.

Widely different is the course of impeded flow of the blood along the veins. An individual vein may be compressed, and the flow of blood along its course, and its return from the organ in which it originates, is impeded ; the capillary vessels, or, as I would term them, the methæmatous channels, are gorged and congested, and the arteries become rigid and throb.

This state of things is conceivable. But this is not all. It is of the most frequent and daily occurrence. And here again I beg to adduce a view of the subject which I believe to have been overlooked. *It is a newly discovered principle in pathology.*

We have only to watch the condition of the platysma-myoides, and of the external jugular vein, to observe that the contraction of that muscle is frequently spasmodic, and the dilatation of the vein, and of the veins which lead to it, a constant effect.

Spasmodic action of the muscle, then, may tend to the obstruction of the course of a vein, and to consequent congestion of its roots, and of the blood channels, to which it serves as a drain.

I must be allowed to repeat, that this action of the muscles must be abnormal and spasmodic, for, as I have said, normal muscular action does not produce this effect. All the muscles of the neck, for example, may be summoned into normal action of the most energetic kind, as in carrying a heavy load on the head, without producing this effect. But let this action be abnormal, irregular, spasmodic, violent therefore, and without equipoise, and a very different result is observed; the subjacent vein may be compressed, and all the consequences of such compression may occur, viz: distention of its roots, and of the blood-channels, placed intermediately between them and the corresponding branches of arteries, which latter become rigid and throbbing.

Another pathological principle must be adduced in this place. Let any one observe the eyes, the countenance, the tongue, the neck, the hands, &c., in spasmodic disease. They will be satisfied that there is no individual muscle, no series of muscles, which may not be excited into abnormal and violent, because spasmodic action.— There is consequently no vein within the influence of such action which may not be compressed. As a further consequence, there is no organ which delivers up its blood to such vein, which may not be the seat of congestion, and, if I may so express myself, of the apoplectic state.

Now, in the region of the neck there are *four* veins of vast importance in this point of view; these are—

1. The External } Jugulars;

2. The Internal }

3. The Vertebral;

4. The Subclavian.

Now, the external jugular is compressed by the action of the platysma-myoides; the internal jugular, by that of the cleido-mastoid and the omo-hyoid muscles; the vertebral and the subclavian veins may be compressed, and the course of the blood in them impeded or arrested, by the spasmodic action of the scaleni, the subclavius, the pectoralis minor, &c.

To show the influence of abnormal contraction of the muscles on the subjacent or adjacent veins, I may mention the fact, that even the pulse of the artery at the wrist may be stopped by violent voluntary action of the pectoralis minor, and other muscles similarly seated.

The compression of each of these veins induces its own peculiar effect.

The external jugular is frequently compressed by the action of the platysma-myoides, the effect of emotion, and blushing is the consequence; in other cases, the superficial veins of the neck, face, forehead, temples, &c., are seen to become tumid, the face to flush, and the temporal arteries to throb.

The internal jugular may be compressed without any obvious external sign, its roots being deeply seated. But the brain suffers, and there may be one or more of the varied symptoms of *cerebral* epilepsy—that is, momentary loss of consciousness, affection of the vision, or ringing in the ears.

If the vertebral vein be obstructed, there are some of the symptoms of affection of the medulla oblongata, or of *spinal* epilepsy—that is, laryngismus, strabismus, odaxismus, twisted neck, &c.

Lastly, when the subclavian is compressed, the hand of the patient becomes livid and cold. Such an event I have just witnessed, and, indeed, watched, in a little patient, conjointly with Mr. Hodding. The lividity and its accompanying coldness alternated with return of the natural color and warmth from time to time, as the action of the subclavian (as I suppose) varied.

Some action of this kind also doubtless affects the mamma and the nipple, under the influence of what is called "the draught," and of the erectile excitement of the nipple on the pressure of the lips of the infant.

Other glands may be affected in a similar manner, excited, or arrested, by similar means, especially the salivary.

The subject of impeded venous circulation is not exhausted. It is physiological as well as pathological, though most frequently the latter, and always traceable to emotion, or excitants of a reflex action, and *the Spinal System.* Once more a new field of inquiry opens upon us.

It must be here observed, that compression of the subclavian vein may affect, not the brachial vein only, but, in a secondary manner, the vertebral and the jugulars; and it is to be particularly borne in mind, that the veins of this region are not affected *singly* in the manner I have described, but variously together.

I repeat that, in spite of anything which I may have written, or may write, respecting the action of individual muscles on individual veins in the neck, *it is not by any regular and physiological convulsion, but by irregular ab-*

*normal, and violent* GROUPING *and* CONTRACTION *of the muscles of the neck, that compression of the veins of this region become variously affected with its consequences.*

I have now to evolve a fourth pathological principle in relation to this subject. The actions of the muscles to which I have adverted being one and all spasmodic or convulsive, the effects of this on the veins, and more remotely on the cerebrum, or on the medulla oblongata, are—*paroxysmal.* And this remark leads me to mention, in the most emphatic manner, that not only coma in the apoplectic state, but hemiplegia and partial paralysis, and mania, may, as well as epilepsy itself, be paroxysmal, be dependent on intra-vascular congestion, and exist entirely independent of extra-vascular, or other physical change They may be evanescent, therefore, and so, far, very far, less grave than other forms of these diseases.

I feel that this subject, in all its relations to its causes, its rationale, its prognosis, is of vast importance in medical science and art.

*Emotion* and *causes of reflex action* may induce contraction of the muscles of the neck—*trachelismus.* This may compress the veins of the neck, and induce a condition which may be designated *phlebismus.* This leads to congestion of the intermediate blood-channels and the apoplectic state ; and this, primarily or secondarily, to comatose, to paralytic, to maniacal, to epileptic affections, all having the one characteristic feature—that of paroxysmal and evanescent forms. I imagine that this view of the subject is equally original, important, and extensive. The events of each day's practice prove that these paroxysmal forms of diseases of the nervous system, not formerly viewed as paroxysmal, are extremely frequent. In fact, I believe a new ray of light is being shed on apoplexy, and even on paralysis and on mania, in their varied forms —in a word, on a *whole Class* of paroxysmal diseases.

I would here specially remark, or repeat, that impeded flow of blood along the external jugular is seen, first, in the fullness of this vessel itself; second, in that of the intermediate blood-channels; and third, in that of the temporal artery; and that the effects of compression of the subclavian are seen in the lividity and coldness of the hands and fingers ; but the *arrière* connexions of the internal jugular and of the vertebral veins are more deeply seated, and impeded circulation in these vessels is manifested chiefly by *symptoms*—the symptoms of cerebral and of spinal epilepsy respectively.

I may now ask an important question—What are the exciting causes of trachelismus and its phenomena? And I answer, first, emotion; and second, the excitants of reflex action—new subjects of investigation and study in the science and art of medicine.

I am quite aware that neither the professional nor the public mind—they are, indeed, nearly on a par—are raised sufficiently for views so "rational." But, then, I do not write for the present day; and the day will come—and I shall promote its advent—when medicine will form a Science, based on physiology, and calling in the aid both of theory and of observation. These fountains of science will be viewed as allies, not as opponents, and we shall have our Adamses as well as our Hinds, even in medicine. The great Louis, in devoting himself to *observation*, and rejecting the *dreams* of Broussais, would be the last to oppose just and legitimate *theory*. It is strange that in the nineteenth century we should be gravely urged to look and listen, but not to reason or to think; to observe, and yet not to experiment, not to theorize. Besides, theory, and even hypothesis, leads to observation, by teaching us how and what to observe. The hypothesis of a planet exterior to Uranus led to the detection of Neptune. What reflex actions were noticed formerly? Yet who, except the prejudiced, fail to observe them now?

I here conclude this little sketch, but not without one or two final practical remarks.

There is no degree or form of apoplexy or mania which may not be paroxysmal, and dependent on trachelismus. This is also true of paralysis. One patient suddenly and completely lost the power of articulation at one time, and of writing at another, to recover them after an interval. Another patient lost the power of articulation; a second, the use of the arm, and a third, the use of both arm and leg, yet only for a time.

In most of these cases, but not in all, the paralysis is not only paroxysmal, but more or less combined with spasm—that is, they are not cerebral only, but spinal.

The difference between these paroxysmal forms and the permanent forms of this Class of disease, will now be perceived to be immense. They are especially more curable.

The objects forming this class are far more intimately allied than has been supposed; apoplexy, paralysis, mania, have alike resulted from an epileptic seizure. Minor degrees of the former have occurred from milder degrees of the latter; and even in the entire absence of epileptic symptoms. But to the physiologist there is a bond of union between them all. All may equally, and conjointly or separately, arise from emotion or the excitants of reflex action; from the occasional effects of these inducing contraction of the muscles of the neck, of this on the veins, of this again on the capillary circulation, of this on the condition of the cerebrum, and of the medulla oblongata—whence the Class of paroxysmal, cerebral, and spinal diseases.

Before I conclude I must make one further remark on the structure of the veins of the neck. These are so provided with valves at their conjunction with the subclavian as to cut off the influence of the venous circulation in the fore-arm and arm. Without this provision, each energetic use of the anterior extremity, as when the blacksmith raises his heavy hammer, or strikes with violence on the anvil, would be attended by a blow or shock to the cerebrum, or the medulla oblongata.

One more remark, which I owe to Mr. Hilton, of Guy's. The subclavian artery and vein are protected from the ordinary and normal action of the subclavian muscle by a firm fascia. It is only in its violent abnormal and spasmodic action that the hard and tumid belly of this muscle is made to compress the subjacent vein.

I have, on a former occasion, drawn the attention of the profession to the important distinction between paralysis and spasmo-paralysis. In this paper I have, I think, done a service to my profession by calling its attention to *paroxysmal* paralysis, as well as to other paroxysmal affections of the nervous system. These two forms of nervous affection may be combined in the same individual, or occur separately, and present a subject for careful investigation.

I have also noticed cerebral epilepsy. This affection is nearly allied to the paroxysmal form of apoplexy.

As to paroxysmal mania, it is similar in nature to the mania which succeeds to a fit of epilepsy, only occurring without epileptic phenomena.

The principles of treatment embrace the *prevention* and the actual *remedy*, and consist in avoiding or removing causes—1, of emotion; and 2, of reflex action. I must not on this occasion enter into particulars.

I conclude by the following brief recapitulation of the topics thus briefly discussed for the first time in medical literature:

1. Emotion and Excitants of Reflex Action;
2. Irregular and Inordinate Contraction of the Muscles;
3. Compression of the Veins;
4. Congestion of the Organs en arrière:
5. Cerebral and Spinal Paroxysmal Diseases—viz:
6. Paroxysmal Apoplexy, Paralysis, Mania, Epilepsy, occurring singly or consecutively.—*London Lancet.*

